# Functional Depletion of HSP72 by siRNA and Quercetin Enhances Vorinostat-Induced Apoptosis in an HSP72-Overexpressing Cutaneous T-Cell Lymphoma Cell Line, Hut78

**DOI:** 10.3390/ijms222011258

**Published:** 2021-10-19

**Authors:** Kazuyasu Fujii, Masashi Idogawa, Norihiro Suzuki, Keiji Iwatsuki, Takuro Kanekura

**Affiliations:** 1 Department of Dermatology, Kagoshima University Graduate School of Medical and Dental Sciences, Kagoshima 890-8520, Japan; takurok@m2.kufm.kagoshima-u.ac.jp; 2 Department of Medical Genome Sciences, Research Institute for Frontier Medicine, Sapporo Medical University, Sapporo 060-8556, Japan; idogawa@sapmed.ac.jp; 3 Department of Dermatology, Okayama University Graduate School of Medicine, Dentistry and Pharmaceutical Sciences, Okayama 700-8558, Japan; dermanori@yahoo.co.jp (N.S.); kiwatsuki35@gmail.com (K.I.)

**Keywords:** histone deacetylase inhibitor, cutaneous T-cell lymphoma, HSP72, quercetin, combination therapy

## Abstract

Histone deacetylase inhibitors (HDACis) are one of the therapeutic options for cutaneous T-cell lymphoma (CTCL), but they have limited effects. We previously demonstrated that HSP72 overexpression is associated with chemoresistance to HDACis in lymphoma cells. The purpose of this study was to investigate whether the functional depletion of HSP72 enhances the effect of the HDACi vorinostat. First, we established a stable HSP72-knockdown CTCL cell line and confirmed the influence of HSP72 reduction on the antitumor effects of vorinostat. Next, we studied the effect of quercetin, an inhibitor of HSP72, on the antineoplastic effects of vorinostat. In five CTCL cell lines examined, HSP72 expression was highest in Hut78 cells, and HSP72 knockdown enhanced vorinostat-induced apoptosis in these cells. Low-dose quercetin reduced HSP72 expression, increased HDAC activity, and enhanced vorinostat-induced suppression of Hut78 cell proliferation. A single low dose of quercetin induced G2 arrest and only slightly increased the sub-G1 cell fraction. Quercetin also significantly enhanced vorinostat-induced apoptosis, caspase-3, caspase-8, and caspase-9 activity, and the loss of mitochondrial membrane potential. HSP72 knockdown enhanced vorinostat-induced apoptosis in an HSP72-overexpressing CTCL cell line, and thus, quercetin may be a suitable candidate for combination therapy with vorinostat in clinical settings.

## 1. Introduction

Cutaneous T cell lymphomas (CTCLs) comprise a heterogeneous group of skin-based neoplasms of T-cell origin, with mycosis fungoides (MF) being the most common [1]. Most patients with early-stage MF follow an indolent course. Management of early-stage MF involves more conservative approaches such as skin-directed therapies. In contrast, patients with advanced-stage MF and Sézary Syndrome (SS), a leukemic variant of CTCL, have a poor prognosis with a median survival of less than 5 years [2]. There is currently no curative therapy for advanced MF/SS. Systemic treatments with biological modulators or targeted therapies are recommended, whereas cytotoxic or immunosuppressive chemotherapies are reserved, as chemotherapy shortens the median time until the next treatment in patients with MF/SS [3] and multiagent chemotherapy often induces immunosuppression, which leads to an increased risk of infection [4], one of the most common causes of death in patients with advanced-stage MF/SS [5]. In comparison, interferons and histone deacetylase inhibitors (HDACis) afford longer times to the next treatment than chemotherapy [6].

HDACis are novel medications for the treatment of multiple malignancies, including CTCL. However, their effectiveness is limited, similar to that observed for other anticancer molecular-targeted agents. The objective response rate for HDACis for CTCL is ~30% and the median duration of response is ~8 weeks [7,8,9,10]. Combination therapies with HDACis have been developed. Panobinostat has been used in combination with bortezomib and dexamethasone for patients with multiple myeloma [11]. No established combination therapies with HDACis have been administered for CTCL. However, many attempts, including preclinical trials, have been made to test combinations, such as vorinostat and a phosphoinositide-3 kinase inhibitor [12], vorinostat, interferon-α and extracorporeal photopheresis [13], vorinostat and bexarotene [14], romidepsin and azacytidine [15], romidepsin and lenalidomide [16], vorinostat and etretinate [17], and romidepsin and ponatinib [18], among others.

The search for a predictive marker of sensitivity has been attempted to improve outcomes associated with HDACi treatment for CTCL [19,20,21]. HR23B is recognized as a representative prognostic marker for the therapeutic efficacy of HDACis [19,22], including for CTCL, but not as a target of combination therapy with HDACis. We found that high expression of HSP72, a member of the HSP70 family, is positively correlated with HDACi resistance in lymphoid cell lines [23,24]. Treatment with HDACis has been shown to induce HSP72 expression in various tissues [25,26], including CTCL tumor tissues. In various malignancies, HSP72 is also associated with chemoresistance to a variety of anticancer agents [27,28]. Furthermore, normal tissues usually express a constitutive member of the Hsp70 family, Hsc73, but not Hsp72 [27,29]. Therefore, HSP72 should be a target of combination therapy with HDACis.

Quercetin (3,3′,4′,5,7-pentahydroxyflavone) is a natural flavanol found in many fruits and vegetables, such as broccoli, yellow onions, and apples [30]. It exerts anticancer effects on a variety of cancers, including hematological malignancies [31]. Quercetin has been reported to inhibit the growth of leukemic cells without suppressing normal hematopoiesis [32]. The antitumor effects of quercetin are via the inhibition of various intracellular pathways [33], including the HSP70 pathway. Quercetin has been reported as one of the most effective HSP70 inhibitors [34]. The mechanism through which quercetin suppresses HSP70 expression is not well understood. However, a pathway via heat shock factor 1 (HSF1) has been proposed as one such mechanism. Quercetin is not only effective as a single agent but also potentiates the cytotoxic effect of other chemotherapeutic agents [35]. For example, quercetin enhances the effect of gemcitabine by reducing HSP72 expression [36]. Furthermore, it affects HDAC activity, but its effect on HSP72-overexpressing cells treated with an HDACi is unknown. Here, we investigated the effects of the functional depletion of HSP72 on vorinostat treatment using a stable HSP72-knockdown CTCL cell line.

## 2. Results

### 2.1. HSP72 Expression in CTCL Cell Lines

**Figure 1:**
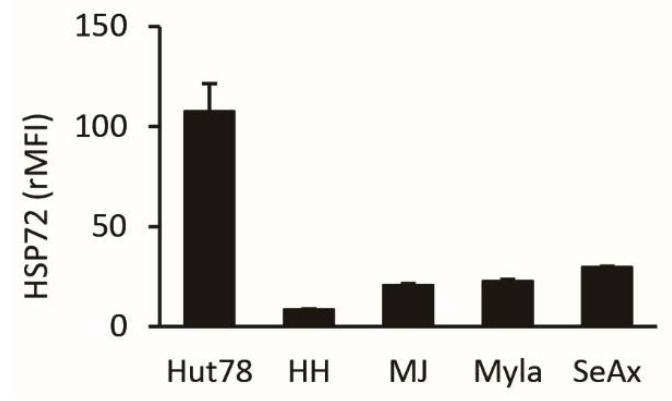
Expression of HSP72 protein in five cutaneous T-cell lymphoma (CTCL) cell lines. Expression of HSP72 in each cell line was described as the relative mean fluorescence intensity (rMFI) + SD (n = 3).

We first assessed the protein expression level of HSP72 in five CTCL cell lines. As shown in Figure 1, it was highest in Hut78 cells (relative mean fluorescence intensity [rMFI]: 107), followed by SeAx (rMFI: 30), Myla (rMFI: 23), MJ (rMFI: 21), and HH (rMFI: 9) cells. Accordingly, we used Hut78 cells in subsequent studies.

### 2.2. Establishment and Characterization of HSP72-Knockdown Hut78 Cells

**Figure 2:**
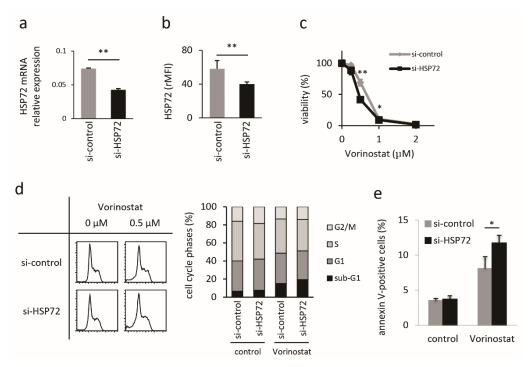
Characterization of HSP72-knockdown Hut78 cells. *HSP72* mRNA expression for MOCK (gray bar) and HSP72-knockdown (black bar) Hut78 cells, normalized to *GAPDH* expression levels ((**a**), n = 2). Flow cytometric analysis of HSP72 protein expression in MOCK (gray bar) and HSP72 knockdown (black bar) cells, showing the relative mean fluorescence intensity (rMFI) + SD ((**b**), n = 3). Proliferation assay with vorinostat using the WST-1 assay kit ((**c**), n = 4). ◆: MOCK, and ■: HSP72 knockdown Hut78 cells. Assessment of the influence of HSP72 knockdown on the antitumor effects of vorinostat. Representative images are shown in the left panels, and the summary is shown in the right panel (**d**). Assessment of apoptosis by annexin-V assays (**e**). * *p* < 0.05, ** *p* < 0.01, by student *t*-test.

Next, we established an HSP72-stable knockdown cell line from Hut78 cells, as described in the Materials and Methods section. *HSP72* mRNA expression was reduced by 40% (Figure 2a), and protein expression was decreased by 20% in knockdown cells (Figure 2b), which were more sensitive to vorinostat than MOCK cells (Figure 2c). IC_50_ values at 24 h of MOCK and HSP72-knockdown cells were 0.66 μM and 0.46 μM, respectively. The propidium iodide flow cytometric assay revealed that the HSP72 knockdown itself did not affect apoptosis and the cell cycle (proportions of sub-G1 cells in MOCK and HSP72-knockdown cells were 6.6 ± 1.3% and 7.6 ± 0.4%, respectively; *p* = 0.45). However, HSP72 knockdown enhanced vorinostat-induced apoptosis (Figure 2d). After a 24 h treatment with 0.5 μM vorinostat, proportions of sub-G1 cells in MOCK and HSP72-knockdown cells were 15.1 ± 1.3% and 19.3 ± 1.5%, respectively (*p* = 0.003). Cell cycle distribution, other than sub-G1, was not affected by HSP72 expression in either untreated or vorinostat-treated cells. The effect of HSP72 reduction on vorinostat-induced apoptosis was confirmed by an annexin V assay (Figure 2e). Annexin V-positive cells in vorinostat-treated MOCK and HSP72-knockdown cells comprised 8.1 ± 1.7% and 11.7 ± 1.1% of cells, respectively (*p* = 0.02).

### 2.3. Quercetin Reduces HSP72 Expression and Enhances HDAC Activity

**Figure 3:**
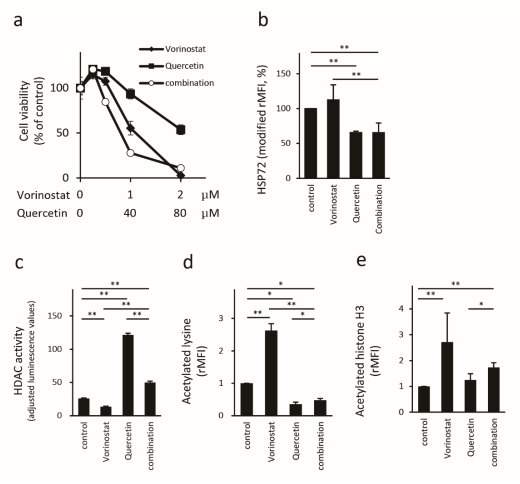
Influence of quercetin on vorinostat-treated Hut78 cells. Cell proliferation was assessed by WST-1 assays ((**a**), n = 4). ◆: treated by vorinostat, ■: quercetin, and ○: both vorinostat and quercetin (1:40). HSP72 protein expression was measured via FACS (**b**). HDAC activity was assessed by the HDAC-Glo™ I/II Assays and adjusted with the CellTiter-Fluor^TM^ Cell Viability Assay (**c**). Acetylated lysin (**d**) and acetylated histone H3 (**e**) were measured via FACS. * *p* < 0.05, ** *p* < 0.01 by Bonferroni *t*-test.

Next, we assessed the anti-proliferative effect of vorinostat in combination with quercetin via the WST-1 assay (Figure 3a). A high dose (80 μM) of quercetin suppressed Hut78 cell proliferation (53.8 ± 5.4%), whereas a low dose (20 μM) did not inhibit cell proliferation (118.6 ± 4.4%). In the same way, high-dose (2.0 μM) vorinostat suppressed cell proliferation (2.9 ± 7.6%), whereas low dose (0.5 μM) vorinostat did not inhibit cell proliferation (107.6 ± 0.7%). However, a combination of low-dose vorinostat and low-dose quercetin inhibited the proliferation of Hut78 cells (84.6 ± 3.0%). The combination of 1 μM vorinostat and 40 μM quercetin synergistically inhibited cell proliferation, compared to that with each agent alone (*p* = 0.0038, two-way ANOVA; vorinostat alone = 55.5 ± 7.6%, quercetin alone = 93.5 ± 5.6%, and combination = 27.9 ± 2.9%). Our results indicated that 0.5 μM vorinostat and 20 μM quercetin were the optimal concentrations for further examination, and this quercetin concentration is available in vivo [37]. Before further examination, we confirmed the effect of these agents on HSP72 expression in Hut78 cells. Vorinostat did not affect HSP72 expression in Hut78 cells, whereas quercetin reduced HSP72 expression, both with single-use (33.5 ± 6.3%) and in combination with vorinostat (42.4 ± 4.8%; Figure 3b). Next, we assessed the effects of quercetin on HDAC activity on lysine and histone H3 acetylation statuses. The single-use of vorinostat reduced HDAC activity (51.8 ± 2.7%; Figure 3c), whereas quercetin enhanced HDAC activity (470 ± 19%) and enhanced the HDAC activity that had been reduced by vorinostat (373 ± 25%, compared to vorinostat treatment). In line with the HDAC activity assay results, vorinostat enhanced lysine acetylation (263 ± 21%), whereas quercetin reduced lysine acetylation (36.1 ± 5.7%), even in the presence of vorinostat (18.3 ± 2.0%, compared to that in vorinostat-treated cells; Figure 3d). Similarly, vorinostat enhanced the acetylated status of histone H3 (283 ± 83%), whereas quercetin treatment did not affect histone H3 acetylation (124 ± 17%). Interestingly, the combination of vorinostat and quercetin enhanced histone H3 acetylation (162 ± 23%; Figure 3e) compared to that in the untreated controls.

### 2.4. Quercetin Enhances Vorinostat-Induced Apoptosis

**Figure 4:**
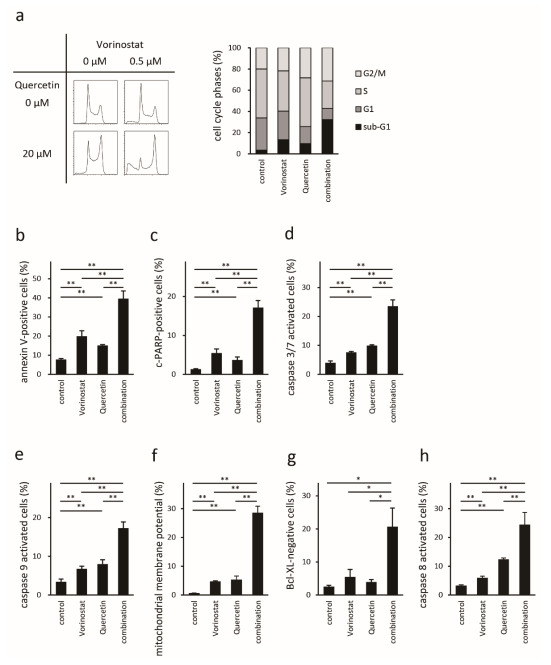
Assessment of the effect of quercetin on the antitumor effects of vorinostat in Hut78 cells. Propidium iodide staining (**a**). Representative images are shown in the left panels and the summary of the analysis is shown as a bar graph in the right panel. Annexin V assay (**b**); cleaved PARP assay (**c**); activated caspase-3/7 (**d**), caspase-9 (**e**), and caspase-8 (**h**) analysis; loss of mitochondrial membrane potential (**f**); bcl-XL expression (**g**) as assessed via FACS. * *p* < 0.05, ** *p* < 0.01 by Bonferroni *t*-test.

Finally, we assessed how quercetin enhanced vorinostat-induced antitumor effects. Cell cycle distribution analysis showed that single-use low-dose vorinostat induced mild apoptosis, whereas single-use quercetin mainly induced G2 arrest in addition to a mild increase in the sub-G1 cell fraction. Nonetheless, quercetin significantly enhanced vorinostat-induced apoptosis. Quercetin-induced G2/M phase cell accumulation was dominant in the combination group (Figure 4a). Annexin V and cleaved-PARP assays confirmed the synergistic effects of vorinostat and quercetin on apoptosis (Figure 4b,c). The percentages of annexin V-positive cells were 7.7 ± 0.5% in the control, 19.9 ± 2.8% in vorinostat-treated cells, 15.1 ± 0.5% in quercetin-treated cells, and 39.6 ± 4.0 in the combination group. The percentages of cleaved-PARP-positive cells were 1.3 ± 0.1%, 5.5 ± 1.1%, 3.7 ± 0.8%, and 17.1 ± 1.8% in the control, vorinostat, quercetin, and combination groups, respectively. We next assessed the effect of quercetin on caspase activity. Single-use vorinostat or quercetin enhanced caspase-3 activation, and the combination of vorinostat with quercetin significantly enhanced caspase-3 activity (Figure 4d). The percentages of caspase-3 activated cells were 4.0 ± 0.7%, 7.6 ± 0.3%, 9.9 ± 0.3%, and 23.6 ± 2.1% in the control, vorinostat, quercetin, and combination groups, respectively. Subsequently, we assessed quercetin-enhanced vorinostat-induced caspase-9 activation (Figure 4e), loss of mitochondrial membrane potential (Figure 4f), and bcl-XL cleavage (Figure 4g). The percentages of cells with active caspase-9, those with depolarization of the mitochondrial membrane potential, and bcl-XL-negative cells were 3.4 ± 0.7%, 0.7 ± 0.02%, and 2.6 ± 0.3% in the control, 6.8 ± 0.7%, 4.8 ± 0.2%, and 5.5 ± 1.8% in the vorinostat group, 8.0 ± 1.2%, 5.4 ± 1.0%, and 3.9 ± 0.6% in the quercetin group, and 17.3 ± 1.6%, 28.6 ± 1.8%, and 20.7 ± 4.6% in the combination group, respectively. These results indicate that quercetin activates the vorinostat-induced mitochondrial apoptosis pathway. Quercetin also enhanced vorinostat-induced caspase-8 activation (Figure 4h). The percentages of caspase-8 activated cells were 3.3 ± 0.2%, 6.0 ± 0.5%, 12.4 ± 0.4%, and 24.4 ± 3.5% in the control, vorinostat, quercetin, and combination groups, respectively. This result indicates that quercetin also augments the vorinostat-induced extrinsic apoptosis pathway. Thus, quercetin enhances vorinostat-induced apoptosis via both intrinsic and extrinsic caspase pathways in Hut78 cells.

## 3. Discussion

Molecular backgrounds related to different sensitivities to HDACis have long been explored to optimize therapeutic strategies. Previously, we demonstrated that the overexpression of HSP72 is associated with resistance to HDACis [23,24]. HSP72 inhibits several components of the apoptotic pathway and confers a survival advantage. HSP72 proteins can render various cancer cell types resistant to numerous cytotoxic antineoplastic drugs. In this report, we first showed that HSP72 reduction enhances chemosensitivity against vorinostat in HSP72-highly expressing cells. In this study, we used Hut78 cells, which expressed the highest level of HSP72 among the five CTCL cell lines examined. Our previous study also showed that Hut78 belonged to the group expressing the highest level of HSP72 compared to that in many non-CTCL hematological cell lines [23]. Stable HSP72-knockdown Hut78 cells were more sensitive to vorinostat than MOCK cells. Furthermore, HSP72 reduction enhanced HDACi-induced apoptosis, which is one of the primary anticancer mechanisms underlying the function of HDACis. These results are in line with our previous report, which demonstrated that stable HSP72-overexpressing Jurkat cells were more resistant than MOCK cells [24]. HDACi treatment was reported to induce the expression of HSP72 [26], whereas vorinostat treatment did not enhance HSP72 expression in Hut78 cells in this study. This result might be due to the high baseline expression level of HSP72 in Hut78 cells or insufficient stimulation time with vorinostat.

In this study, we evaluated the effect of quercetin on the antineoplastic effects of vorinostat. Low-dose quercetin (20 μM), a tolerable concentration for an in vivo model, did not reduce cell proliferation, but reduced HSP72 expression and enhanced the inhibitory effect of vorinostat on cell proliferation. Quercetin is reported to induce caspase-dependent cell death and arrest the cell cycle at the G1/S phase [38] or G2/M phase [39]. In this study, single-agent, low-dose quercetin led to G2/M arrest and rarely induced apoptosis, and quercetin enhanced vorinostat-induced apoptosis through both intrinsic and extrinsic apoptotic pathways with G2/M arrest. Previously, we identified that HSP72 overexpression reduced both the intrinsic and extrinsic caspase pathways activated by vorinostat or valproic acid [24]. Therefore, the reduction in HSP72 mediated by quercetin might have activated these pathways and enhanced apoptosis.

The effects of quercetin on HDAC activities have been reported in numerous studies. Most reports mention that quercetin inhibits HDAC activity [40,41,42,43,44,45], and some of the reports indicated that quercetin enhances HDACi-induced cell death [40,42]. Furthermore, Chan et al. [42] also showed that quercetin enhances HDACi-induced histone H3 and H4 acetylation. The mechanism underlying the potentiation of the antitumor effect of HDACis mediated by quercetin has been postulated to occur by quercetin enhancing the HDAC-inhibitory activity of HDACi. However, we found that quercetin enhanced HDAC activity in Hut78 cells in this study. To our knowledge, there is only one study, reported by Kim et al., mentioning that quercetin can enhance HDAC activity [46]. Although Kim et al. [46] reported that quercetin enhances HDAC activity, they did not investigate the interrelationship between quercetin and HDACis. In this study, we have shown that quercetin synergistically enhances vorinostat-induced apoptosis without decreasing HDAC activity. The acetylation status of lysine was inhibited by quercetin, consistent with increased HDAC activity, whereas single-agent quercetin did not change histone H3 acetylation. Quercetin reduced vorinostat-induced histone H3 acetylation, whereas the histone H3 acetylation status was elevated relative to that of the untreated control. Despite the absence of increased acetylation of histone H3, quercetin enhanced the antitumor effect of vorinostat. We hypothesized that quercetin potentiated the antitumor effect of vorinostat not mainly through the activation of gene transcription through histone acetylation but rather via other novel factors, such as the reduction of HSP72.

## 4. Materials and Methods

### 4.1. Cell Lines and Agent

CTCL cell lines, Hut78, HH, MJ, Myla, and SeAx, were used in this study. The Hut78 cell line was purchased from the Institute of Development, Aging and Cancer, Tohoku University (Miyagi, Japan), and HH and MJ cell lines were purchased from the American Type Culture Collection (Bethesda, MD, USA). Myla and SeAx cell lines were obtained from Prof. R. Dummer (University of Zurich, Zurich, Switzerland). The cells were cultured at 37 °C in 5% CO_2_. For stable knockdown of HSP72, cells were transfected with custom-made iLenti siRNA (scramble siRNA and siRNA against HSP72: GTGTCAAGAGGTCATCTCG [47]) plasmid vectors (Applied Biological Materials, Vancouver, Canada) using Lipofectamine 2000 (Invitrogen, Carlsbad, CA, USA), according to the manufacturer’s instructions. Forty-eight hours post-transfection, stable clones were selected using puromycin. Quercetin was obtained from Sigma-Aldrich (St. Louis, MO, USA) and vorinostat was purchased from Merck (Whitehouse, NJ, USA).

### 4.2. Assessment of HSP72 Expression

*HSP72* mRNA expression was confirmed using real-time RT-PCR, and the ABI 7300 Real-Time PCR system (Applied Biosystems, Foster City, CA, USA) as previously described [24]. Total RNA was isolated using an RNeasy Mini kit (Qiagen, Hilden, Germany) and cDNA synthesis was performed using the SuperScript First-Strand Synthesis system (Invitrogen) according to the manufacturer’s protocols. Next, qRT-PCR was performed using the SYBR Green PCR Master Mix (Applied Biosystems) and an ABI 7300 instrument. RT-PCR reactions contained synthesized cDNA and TaqMan Gene Expression Assay components (Hs00359147_s1 for HSP72 and Hs99999905_m1 for glyceraldehyde 3-phosphate dehydrogenase [*GAPDH*]; Applied Biosystems). *HSP72* expression in each sample was normalized to that of *GAPDH*. HSP72 protein expression was examined by flow cytometry. Briefly, cells were fixed with methanol and permeabilized with phosphate-buffered saline (PBS)-T (PBS containing 0.1% triton X-100). Cells were then washed with PBS, stained with either anti-HSP72-FITC (C92F3A-5; Enzo Life Sciences, Farmingdale, NY, USA) or isotype control antibody, and analyzed by flow cytometry.

### 4.3. Proliferation Assays

Cellular proliferation was measured using a WST-1 assay kit (Roche Diagnostics GmbH, Mannheim, Germany) according to the manufacturer’s instructions. Briefly, cells were seeded at ~1 × 10^4^ cells/well in a 96-well plate and then treated for 24 h with vorinostat, quercetin, or both. Next, WST-1 cell proliferation reagent was added to each well, and the cells were incubated for a further 4 h at 37 °C. The absorbance at 450 nm was then measured using a Multiskan FC microplate reader (Thermo Fisher Scientific, Rockford, IL, USA). The absorbance at a reference wavelength of 690 nm was measured simultaneously.

### 4.4. Assessment of HDAC Activity and Acetylation Status

HDAC enzymatic activity was measured using the HDAC-Glo™ I/II Assays (Promega, Madison, WI, USA) according to the manufacturer’s protocol. HDAC activities were adjusted using the CellTitet-Fluor^TM^ Cell Viability Assay (Promega). Luminescence and fluorescence were measured using a GloMax Discover Multimode Microplate Reader (Promega). The acetylation status of lysine or histone H3 was assessed by flow cytometry using phycoerythrin (PE)-conjugated anti-acetyl-lysine antibody (15G10; BioLegend, San Diego, CA, USA), Alexa Fluor^®^ 488-conjugated anti-acetyl-histone H3 antibody (C5B11; Cell Signaling Technology (CST), Beverly, MA, USA), or respective isotype control antibodies.

### 4.5. Analysis of Cell Cycle and Apoptosis

To measure cellular DNA content, cells were fixed with 70% ice-cold methanol. After the washing step, cells were stained with FxCycle^TM^ PI/RNAse staining solution (Invitrogen, Carlsbad, CA, USA) for 15 min at room temperature and analyzed by flow cytometry. Apoptosis was also assessed using an Annexin V-PE Apoptosis Detection Kit I (BD Biosciences, San Jose, CA, USA) using a flow cytometer. Expression of poly (ADP-ribose) polymerase (PARP) cleavage and bcl-XL were measured with a PE-conjugated cleaved PARP (ASP214) XP^®^ antibody (D64E10; CST) and PE-conjugated Bcl-XL antibody (7B2.5, Beckman Coulter, Miami, FL, USA), respectively.

### 4.6. Caspase Activity Assays

Caspase-3/7, caspase-8, and caspase-9 activities were assessed using the FAM-FLICA^®^ caspase-3/7, caspase-8, or caspase-9 assay kits (Immunochemistry Technologies, Bloomington, MN, USA) according to the manufacturer’s instructions. After stimulation under the indicated conditions, cells were incubated with each FAM-FLICA^®^ solution for 1 h with 5% CO_2_ at 37 °C. Then, the cells were washed twice with 1× apoptosis wash buffer and analyzed via flow cytometry.

### 4.7. Assessment of the Mitochondrial Membrane Potential (MMP)

Changes in the MMP at the single-cell level were investigated using the lipophilic cation JC-1 (Cayman Chemical, Ann Arbor, MI, USA). Treated samples were incubated with the JC-1 probe at a final concentration of 2.5 µg/mL for 30 min and then immediately examined via flow cytometry.

### 4.8. Flow Cytometric Analysis

Samples were analyzed using a BD Accuri C6 flow cytometer (BD Biosciences, San Jose, CA, USA), and data were analyzed using FlowJo software (Tree Star, Ashland, OR, USA). The rMFI was calculated by dividing the mean fluorescence intensity (MFI) of the test antibody by the MFI of an isotype-matched control antibody. Cell cycle analysis was conducted using the Dean-Jett Fox model. All samples were at least tested in triplicate.

### 4.9. Statistics and Data Analysis

Results are expressed as means ± SDs. A Student’s *t*-test or two-way analysis of variance (ANOVA) was used to statistically evaluate differences among treatments. When a difference was found by two-way ANOVA, a post hoc test using the Bonferroni method was performed. *p* < 0.05 was considered significant.

## 5. Conclusions

In conclusion, we found that HSP72 reduction enhances the sensitivity of vorinostat in Hut78 cells, an HSP72-highly expressing CTCL cell line, and that quercetin enhanced the antitumor effect of vorinostat accompanied by the suppression of HSP72 expression in an in vitro model. HSP72 is a possible target of HDACis, and quercetin is a promising candidate for combination therapy with vorinostat in patients with CTCL.

## Data Availability

The data presented in this study are available on request from the corresponding author.
